# Low dose lofexidine for medically directed outpatient opioid tapering in adults with chronic pain: a prospective case series

**DOI:** 10.1186/s13256-023-04309-x

**Published:** 2024-01-17

**Authors:** Megan M. Ellis, Nathan D. Eberhart, Nafisseh S. Warner, W. Michael Hooten

**Affiliations:** Department of Anesthesiology and Perioperative Medicine, Mayo School of Graduate Education, Mayo Clinic, 200 First St SW, Charlton 1-145, Rochester, MN 55905 USA

**Keywords:** Lofexidine, Opioid withdrawal, Chronic pain

## Abstract

**Background:**

In adults with chronic pain, mild-to-moderate withdrawal symptoms during medically directed opioid tapering in the outpatient setting may not be accompanied by hypertension or tachycardia. This clinical scenario could limit the use of lofexidine at dosages reported in clinical trials of opioid withdrawal precipitated by abrupt opioid discontinuation. Thus, the primary aim of this prospective case series is to describe the use of low dose lofexidine for opioid withdrawal in patients with chronic pain undergoing medically directed opioid tapering in an outpatient setting.

**Methods:**

Six patients (white 5, Latino 1) admitted to an outpatient interdisciplinary pain rehabilitation program met inclusion and exclusion criteria. Patients self-selected to undergo medically directed opioid tapering, and the medication the patients were prescribed upon admission was used in the taper schedule. Upon initiation of the opioid taper, patients received 0.18 mg of lofexidine every 6 hours.

**Results:**

Five of the six patients were women, and the median morphine milligram equivalents at baseline were 36.9. The median taper duration was 15 days, and the median duration of lofexidine administration was 14 days. Withdrawal scores were mild throughout the taper in four patients, and two patients with fibromyalgia experienced single episodes of moderately severe withdrawal symptoms at the median morphine milligram equivalent midpoint of the taper. No hypotension or sustained bradycardia were observed, and no adverse effects related to lofexidine were reported.

**Conclusion:**

The observations from this prospective case series suggest that low-dose lofexidine may be a feasible adjunct medication to attenuate withdrawal symptoms in adults with chronic pain undergoing outpatient opioid tapering.

## Introduction

Long-term exposure to opioids alters noradrenergic function in the locus coeruleus through an adenylate cyclase-mediated pathway [1, 2]. During states of opioid deprivation, norepinephrine release increases, which contributes, in part, to the clinical syndrome of opioid withdrawal [3, 4]. The pathophysiological links between endogenous opioids and noradrenergic systems provide the pharmacological basis for using α2-adrenergic receptor agonists to treat signs and symptoms of opioid withdrawal.

The most prevalent subtypes of α2-adrenergic receptors in the locus coeruleus are the α2A and α2C receptors [3–5]. Located in both the pre- and postsynaptic regions, the α2A receptor inhibits norepinephrine release in response to maximum sympathetic activation, while postsynaptic α2C receptors modulate basal norepinephrine release [4–6]. Clonidine is the most widely used α2 agonist for opioid withdrawal, but hypotension and bradycardia can be treatment limiting adverse effects [7]. Lofexidine, which is approved by the US Food and Drug Administration for opioid withdrawal, is a structural analogue of clonidine. In clinical trials, treatment limiting adverse effects, including hypotension and bradycardia, in adults receiving lofexidine for opioid withdrawal are lower compared with clonidine. [8–10]

Despite proven efficacy and a favorable cardiovascular side effect profile, lofexidine use for the management of opioid withdrawal in adults with chronic pain undergoing medically directed tapering in an outpatient setting has not been previously reported. This knowledge gap is important because withdrawal symptoms during tapering may not be accompanied by hypertension or tachycardia, which could limit lofexidine use at dosages reported in clinical trials of opioid withdrawal precipitated by abrupt discontinuation. Thus, the primary aim of this prospective case series is to describe the use of low-dose lofexidine for opioid withdrawal in patients with chronic pain undergoing medically directed opioid tapering in an outpatient setting.

## Methods

This research project was approved by the Mayo Foundation Institutional Review Board and patients provided signed informed consent prior to study participation.

### Patients

The case series is composed of six patients (white 5, Latino 1) admitted to an outpatient interdisciplinary pain rehabilitation program from August 2020 to February 2021. Inclusion criteria included: (1) age ≥ 18 years, (2) daily opioid use ≥ 20 morphine milligram equivalents (MMEs), and (3) chronic noncancer pain of at least 6 months in duration. Exclusion criteria included: (1) nondaily use of opioids and (2) use of tramadol.

### Clinical setting

The clinical setting has been previously described [11]. The outpatient interdisciplinary pain rehabilitation program (PRP) spanned a 3-week period during which patients were involved in 8 hours of consecutive treatment daily for 15 consecutive working days. A cognitive behavioral model served as the basis of treatment, and the primary goal was restoration of physical and emotional functioning. The treatment protocol of the PRP included daily educational group sessions related to optimizing self-management of pain intensity, depressive symptoms, pain-related anxiety, and psychosocial stressors. Other components included daily aerobic and strengthening exercises led by a physical therapist, occupational therapy, elimination of pain behaviors, and individualized recommendations for activity moderation.

### Opioid tapering

Prior to admission, all patients self-selected to undergo medically directed opioid tapering. The dose and frequency of daily opioid use were determined by self-report and review of pharmacy records. The admission opioid dose was converted to daily morphine milligram equivalents (MMEs) using an equianalgesic conversion software program. Consistent with the established treatment protocols of the rehabilitation program, the opioid medication the patients were prescribed upon admission was used in the taper schedule [12, 13]. Due to the broad range of opioid medications, prescribed dosages, and unique patient clinical characteristics, tapers were individually tailored by the physician staff of the pain rehabilitation program. Predetermined taper schedules were not used, and adjunct medications including benzodiazepines, anticonvulsants (for example, gabapentin and pregabalin), muscle relaxants, or sedative hypnotics were not concomitantly initiated to manage withdrawal symptoms. Patients were assessed daily by medical staff for signs and symptoms of opioid withdrawal. Heart rate and blood pressure were monitored daily and more frequently if clinically indicated.

### Lofexidine dosing

Upon initiation of the opioid taper, all patients received 0.18 mg of lofexidine every 6 hours. In the absence of a definition for low-dose lofexidine, the rationale for using the aforementioned dose and dosing schedule was based on the clinical scenario, previously reported doses and adverse effects, and the pharmacokinetics of lofexidine. First, in clinical trials of abrupt opioid discontinuation, 10–30% of subjects experienced hypotension or bradycardia at lofexidine dosages of 0.54 mg and 0.72 mg every 6 hours. [14, 15] The severity of withdrawal symptoms during medically directed tapering of lower opioid dosages is expected to be less severe compared with withdrawal symptoms accompanying abrupt discontinuation. Thus, it was reasoned that a dose of 0.18 mg would mitigate the risk of hypotension and bradycardia in this group of patients who were anticipated to have mild symptoms of opioid withdrawal. Second, for lofexidine, the average time to maximum concentration is 3.3 hours, the elimination half-life is 11 hours, and the time to steady state is 2 days [16]. Thus, based on the pharmacokinetic profile of the lofexidine [16] and frequency of use in previous clinical trials [14, 15], it was reasoned that dosing every 6 hours would optimize the therapeutic potential of the drug for mild symptoms of opioid withdrawal.

### Urine drug testing

Upon admission and dismissal, definitive urine drug testing (UDT) using liquid chromatography/mass spectroscopy was performed to confirm the status of opioid use and screen for substances of abuse.

### Measurements

#### Patient characteristics

Upon admission, clinical data were collected, including age, sex, marital status, smoking status, years of education, duration of pain, opioid use, and daily substance use (including alcohol and cannabis). Gabapentin and pregabalin use was assessed due to the potential favorable effects of these drugs on opioid withdrawal [17].

#### Pain intensity

Pain intensity was assessed using the verbal rating scale (VRS), where 0 indicates no pain and 10 indicates the most intense possible pain. The validity of the VRS has been established. [18]

#### Pain Catastrophizing Scale

The Pain Catastrophizing Scale (PCS) is a 13-item self-report questionnaire that assesses negative emotions and cognitions associated with actual or anticipated pain experiences [19]. The reliability and validity of the PCS have been established [20, 21]. The PCS scores range from 0 to 52, where higher scores indicate greater levels of negative emotions and cognitions associated with pain experiences.

#### Clinical Opiate Withdrawal Scale

The Clinical Opiate Withdrawal Scale (COWS) is an 11-item questionnaire that assesses the severity of acute opioid withdrawal [22]. The validity of the COWS has been established [23]. Scores range from 0 to 47, and higher scores indicate the presence of more severe withdrawal symptoms. Based on the COWS score, withdrawal symptom severity is categorized as mild (≤ 12), moderate (13–24), moderately severe (25–36), and severe (> 36).

### Data summary

Clinical and demographic characteristics were summarized. Median values and the interquartile range (IQR), calculated as the difference between the 75th and 25th quartiles, were reported for all continuous variables, and frequency and proportion were reported for noncontinuous variables.

## Results

### Demographics and clinical characteristics

Table 1 presents the clinical and demographic information. The results of the admission UDT were consistent with the prescribed opioid for all patients. Five of the six patients were women and the median MME at baseline was 36.9. The median taper duration was 15 days and the median duration of lofexidine administration was 14 days. Successful completion of all opioid tapers were confirmed by UDT at program dismissal, except for patient 6, who completed the taper independent of daily program participation, and patient 3, due to a laboratory ordering error.

**Table 1:** Demographic and clinical characteristics

	Patient 1	Patient 2	Patient 3	Patient 4	Patient 5	Patient 6	Data summary
Pain diagnosis	Bilateral leg	Sacral spine	Fibromyalgia	Fibromyalgia	CRPS^a^	Thoracic spine	
Age	57	66	40	38	48	60	52.5 (17.3)^b^
Sex	Male	Female	Female	Female	Female	Female	83.3% female
Marital status	Married	Single	Married	Married	Married	Married	83.3% married
Smoking status	Never	Former	Former	Current	Former	Current	33% current smoker
Education (years)	15	17	15	14	19	15	15 (1.5)^b^
Pain duration (years)	20	11	9	12	2	6	10 (5.0)^b^
Pain score^c^	5	7	7	7	7	8	7 (0.0)^b^
PCS^d^	34	17	35	12	38	39	34.5 (16.0)^b^
Baseline MME^e^	37.5	35.0	48.0	22.5	45.0	36.3	36.9 (7.8)^b^
Taper duration (days)	15	16	19	15	12	15	15.0 (0.8)^b^
Lofexidine duration (days)	14	15	18	14	11	13	14.0 (1.5)^b^

^a^CRPS, complex regional pain syndrome; ^b^median (interquartile range); ^c^verbal rating scale; PCS, ^d^Pain Catastrophizing Scale; ^e^MME, morphine milligram equivalent

### Case series

**Fig. 1:**
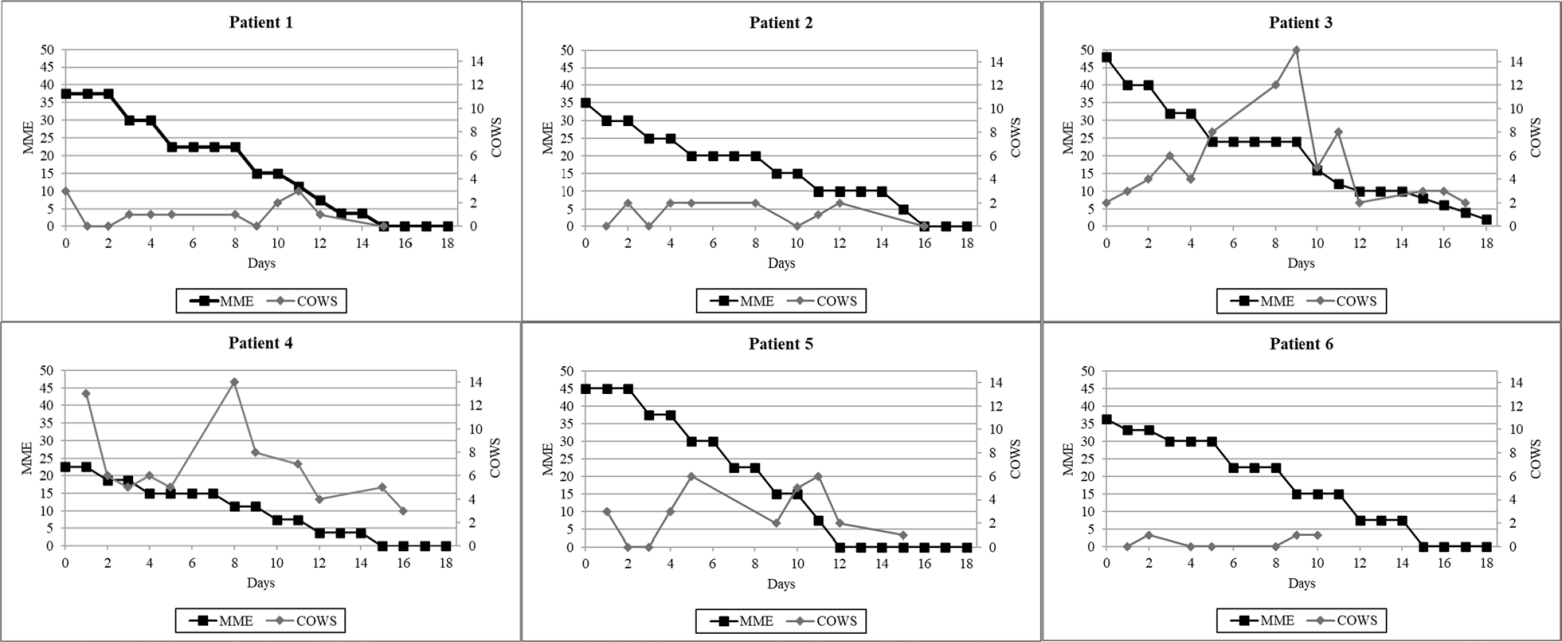
Reduction in morphine milligram equivalents (MMEs) and Clinical Opiate Withdrawal Scale (COWS) for each patient undergoing medically directed opioid tapering

**Fig. 2:**
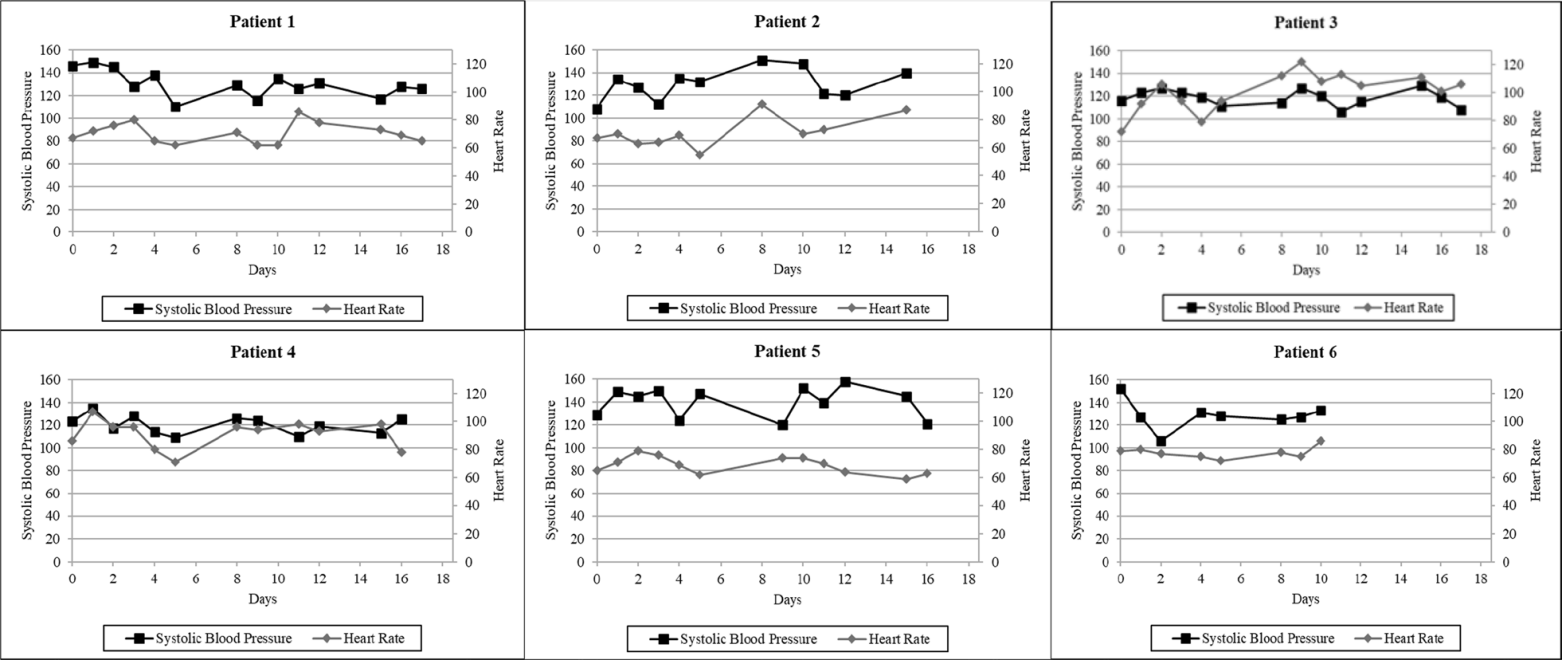
Systolic blood pressure and heart rate of each patient throughout the duration of the medically directed opioid taper

Patient 1 is a 57-year-old male with chronic bilateral anterior thigh pain due to a history of inflammatory myositis (Table 1). Upon admission, he was receiving up to five oxycodone 5 mg tablets daily. He was placed on 0.18 mg of lofexidine every 6 hour,s and the flexible oxycodone taper was completed on day 15 of the PRP. Mild elevation in COWS scores were observed on days 10 and 11, but the COWS scores returned to baseline on day 12 (Fig. 1). No clinically significant changes in heart rate or blood pressure were observed (Fig. 2), and no lofexidine-related adverse effects were reported.

Patient 2 is a 66-year-old female with chronic coccygeal pain (Table 1). The admission UDT detected delta-9 carboxy-tetrahydrocannabinol (THC) at a concentration of 68 ng/ml, and abstinence from cannabis was recommended throughout the duration of the RPR. Upon admission, she was receiving 10 mg/325 mg of hydrocodone/acetaminophen (total daily dose hydrocodone 35 mg). She was placed on 0.18 mg of lofexidine every 6 hours, and the flexible hydrocodone taper was completed on day 16 of the PRP. The highest COWS score was 3 (Fig. 1). Abstinence from cannabis was supported by the UDT results at RPR dismissal where THC was not detected. No clinically significant changes in heart rate or blood pressure were observed (Fig. 2), and no lofexidine-related adverse effects were reported.

Patient 3 is a 40-year-old female with fibromyalgia (Table 1). Upon admission, she was receiving 4 mg of hydromorphone three times daily. She was placed on 0.18 mg of lofexidine every 6 hours and the flexible hydromorphone taper was completed on day 19 of the RPR. Withdrawal symptoms intensified over a 2-day weekend (Fig. 1). On day 8, which was the ensuing Monday, lofexidine was increased to 0.36 mg every 6 hours. Over the next 24-hours, the severity of withdrawal symptoms improved. The increased lofexidine dose was continued for 6 days after which the dose was reduced to 0.18 mg every 6 hours and then discontinued on day 18. Intermittent episodes of tachycardia consistent with mild opioid withdrawal were observed, but no sustained elevations in systolic blood pressure were recorded (Fig. 2). No lofexidine-related adverse effects were reported.

Patient 4 is a 38-year-old female with fibromyalgia (Table 1). The admission UDT detected THC at a concentration of > 500 ng/ml, and abstinence from cannabis was recommended throughout the duration of the RPR. Upon admission, she was receiving up to three tablets of 7.5 mg/325 mg of hydrocodone/acetaminophen daily and 900 mg of gabapentin three times daily. She was placed on 0.18 mg of lofexidine every 6 hours, and the flexible hydrocodone taper was completed on day 18 of the RPR. A spuriously elevated COWS score was documented at baseline prior to initiating the opioid taper (Fig. 1). Withdrawal symptoms intensified over a 2-day weekend. On day 8, the following Monday, the peak COWS score was due primarily to elevated ratings on the anxiety or irritability and restlessness subscales. In addition to tapering opioids, she abruptly discontinued cannabis upon admission. In the absence of sustained elevations in blood pressure or heart rate (Fig. 2), the medical staff concluded that acute cannabis withdrawal, which is characterized by anxiety and irritability, was contributing to the overall clinical scenario. The lofexidine dose was not increased, and withdrawal symptoms improved using the cognitive behavioral techniques of the pain rehabilitation program. Abstinence from cannabis was supported by the UDT results, where the THC concentration decreased to 96 ng/ml at program dismissal. No lofexidine-related adverse effects were reported.

Patient 5 is a 48-year-old female with complex regional pain syndrome type 1 (Table 1). Upon admission, she was receiving 10 mg/325 mg of oxycodone/acetaminophen three times daily and 300 mg of gabapentin three times daily. She was placed on 0.18 mg of lofexidine every 6 hours, and the flexible hydrocodone taper was completed on day 12 of the RPR. Mild elevations in COWS scores were observed on days 5, 10, and 11, but the lofexidine dose was not increased (Fig. 1). No clinically significant changes in heart rate or blood pressure were observed (Fig. 2), and no lofexidine-related adverse effects were reported.

Patient 6 is a 60-year-old female with chronic thoracic spine pain. Upon admission, she was receiving 15 mg of immediate-release morphine two to three times daily (Table 1). The average MME was 36.3 for the 7-day period prior to admission. She was placed on 0.18 mg of lofexidine every 6 hours, and the flexible morphine taper was completed on day 15 of the RPR. On day 10, she was unable to fully participate in the physical and occupational therapy components of the program, but the opioid taper was successfully completed independent of daily program attendance (Fig. 1). No clinically significant changes in heart rate or blood pressure were observed (Fig. 2), and no lofexidine-related adverse effects were reported.

## Discussion

In this prospective case series involving adults with chronic pain tapering lower dosages of opioids, mild symptoms of opioid withdrawal were observed with low dose lofexidine, except for two episodes of moderately severe withdrawal symptoms. No episodes of hypotension or sustained bradycardia were observed, and no treatment limiting adverse effects were reported.

The two isolated episodes of moderately severe withdrawal occurred at the midpoint of the opioid taper in two patients with fibromyalgia. Although cannabis withdrawal [24] may have worsened withdrawal symptoms in one patient with fibromyalgia, alternations in endogenous opioid activity have been previously observed in patients with fibromyalgia. For example, patients with fibromyalgia have increased cerebrospinal fluid levels of endogenous opioids and reduced μ-opioid receptor binding availability, which has been associated with increased levels of pain [25, 26]. Although patients with fibromyalgia undergoing opioid tapering at our outpatient rehabilitation program experience significant improvements in physical and emotional functioning [27], altered opioid activity could potentiate withdrawal symptoms and increase the intensity of withdrawal hyperalgesia. [13]

The lofexidine dose used in this case series of adults with chronic pain warrants comparison with dosing strategies and patient populations reported in two large clinical trials. In a 7-day inpatient randomized, double-blind, placebo-controlled trial, adults with opioid withdrawal after abrupt opioid discontinuation were randomized to placebo, 0.54 mg of lofexidine every 6 hours, or 0.72 mg of lofexidine every 6 hours. [14] The COWS scores peaked at 6–7 on trial day 2, and both lofexidine dosing schedules were superior to the placebo in attenuating opioid withdrawal. However, in both lofexidine groups, 30% of subjects experienced hypotension. Additionally, 23% experienced bradycardia in the 0.54-mg dose group compared with 31% in the 0.72 mg group [14]. In a similarly designed 8-day inpatient trial, subjects were randomized to placebo or 0.8 mg of lofexidine HCL (equivalent to 0.72 mg of lofexidine) every 6 hours. [15] Lofexidine was superior to the placebo, and peak withdrawal scores occurred on study day 1. However, hypotension and bradycardia occurred in 25% and 10% of subjects, respectively, in the lofexidine group, and four subjects required additional inpatient monitoring. [15]

This study has limitations. First, generalization of the reported clinical observations to other groups of patients with chronic pain undergoing opioid tapering are limited by the sample size and methodology of the case series design. However, the findings provide preliminary data about the feasibility of using low-dose lofexidine in this patient population. Second, broader safety claims about low-dose lofexidine cannot be generalized beyond what was observed in this case series.

## Conclusions

The observations from this prospective case series suggest that low-dose lofexidine may be a feasible adjunct medication to attenuate withdrawal symptoms in adults with chronic pain undergoing outpatient opioid tapering.

## Data Availability

All data generated and analyzed during this study are included in this published article.

## Abbreviations

MME: Morphine milligram equivalent
UDT: Urine drug testing
VRS: Verbal rating scale
PCS: Pain Catastrophizing Scale
COWS: Clinical Opiate Withdrawal Scale
IQR: Interquartile range
THC: Delta-9 carboxy-tetrahydrocannabinol
